# Real World Evidence on Second-Line Palliative Chemotherapy in Advanced Pancreatic Cancer

**DOI:** 10.3389/fonc.2020.01176

**Published:** 2020-07-27

**Authors:** Emma Gränsmark, Nellie Bågenholm Bylin, Hakon Blomstrand, Mats Fredrikson, Elisabeth Åvall-Lundqvist, Nils O. Elander

**Affiliations:** ^1^Department of Oncology, Kalmar County Hospital, Kalmar, Sweden; ^2^Department of Oncology, Linköping University, Linköping, Sweden; ^3^Department of Biomedical and Clinical Sciences, Linköping University, Linköping, Sweden; ^4^Department of Clinical Pathology, Linköping University, Linköping, Sweden; ^5^Forum Östergötland, Linköping University, Linköping, Sweden

**Keywords:** pancreatic cancer, palliative therapy, cancer chemotherapy, prognostic factors, CA-19-9 antigen, human plasma albumin

## Abstract

**Background:** The outcome and tolerability of palliative second line chemotherapy for advanced pancreatic cancer (APC) in real life patients are largely unknown. Prognostic parameters for risk stratification and treatment guidance are lacking.

**Materials and Methods:** A population based multicenter retrospective cohort study was conducted, covering all APC patients who received palliative second-line chemotherapy between 2011 and 2018 at any cancer center in the South East Region of Sweden. Primary outcome was overall survival after second-line therapy (OS2). Time to treatment failure after second-line therapy (TTF2), hematological toxicity, and unplanned hospitalizations were key secondary outcomes. A number of baseline potentially prognostic parameters were assessed.

**Results:** A total of 509 patients received first-line palliative chemotherapy, and of these 167 (33%) received at least one dose of second-line therapy and formed the final study population. Median OS2 was 5.2 months (95% CI = 4.7–5.7) and median TTF2 was 1.9 months (1.5–2.2). OS2 and TTF2 were similar regardless regimen, including comparison of the two most common regimens (fluoropyrimidine monotherapy vs. fluoropyrimidine/oxaliplatin doublet). Multivariate analysis revealed that normal plasma albumin (≥35) and serum CA-19-9 above median (>1,550) were independent predictors for OS2 (HR = 0.21, *p* < 0.001 and HR = 2.03, *p* = 0.009) and TTF2 (HR = 0.22, *p* < 0.001 and HR = 2.03, *p* = 0.01), while ECOG performance status >1 was predictive for TTF2 (HR = 2.05, *p* = 0.032). Grade 3–4 hematological toxicity was registered in 17 patients (10%). 50 (30%) had at least one event of hospitalization.

**Conclusion:** The real world outcome of second line palliative chemotherapy for refractory APC remains dismal. Baseline plasma albumin, serum CA-19-9, and performance status emerge as key prognostic factors, and should be further studied as tools for individualized treatment decisions.

## Introduction

In spite of recent therapeutic advances, the long term prognosis in patients with advanced pancreatic cancer (APC) remains dismal, with expected 5-year overall survival (OS) <5% (1, 2). Randomized clinical trials have shown a survival benefit for palliative chemotherapy combination regimens such as FOLFIRINOX and gemcitabine plus nab-paclitaxel (Gem/NabP), compared to the previous gold standard gemcitabine monotherapy, in patients with APC and good performance status (3, 4). Despite these advances in first-line therapy, virtually all patients will experience tumor progression within a limited period of time, and the need for effective and well-tolerated second-line therapies therefore remains high. While limited evidence suggests that second-line treatment in patients with APC and preserved performance status is feasible and may offer a survival benefit (5–7), it is unclear to what degree treatment advances reported in controlled clinical studies are evident when applied to unselected patients and translated into the real world context.

To date, there are three completed randomized clinical phase III trials reporting data on different regimens in the second-line setting. In the CONKO-003 study, 168 patients with gemcitabine refractory APC were randomized to second-line treatment with folinic acid and fluorouracil (FF) or oxaliplatin plus FF (OFF). Median overall survival from start of second-line treatment (OS2) was 2.6 months longer in the OFF multi-drug arm compared to FF (5.9 vs. 3.3 months) (6).

In contrast, the PANCREOX study including 108 patients reported inferior OS2 when adding oxaliplatin to folinic acid and fluorouracil (mFOLFOX6) compared to FF (median OS2 6.1 vs. 9.9 months) (7). Although the study populations in CONKO-003 and PANCREOX were similar, there were differences in dosing schedules and post-progression treatment which may have contributed to the divergent results.

In the 3-armed NAPOLI-1 study, 417 patients with metastatic pancreatic cancer, previously treated with gemcitabine based therapy, were randomized to nanoliposomal irinotecan monotherapy, FF, or nanoliposomal irinotecan plus FF. Median OS2 in the combination multi-drug arm was slightly longer (6.1 months) than in the FF (4.2 months) and irinotecan monotherapy arms (4.9 months) (5). While the combination of nanoliposomal irinotecan and FF has gained FDA approval in the United States, regulatory authorities in many European countries discourage from the use of nanoliposomal irinotecan in the public funded health care due to its limited additional value and negative outcomes in cost-benefit analyses (8, 9).

A recent systematic review on second line chemotherapy following gemcitabine based first line regimens in APC concludes that the design and conduct of these three trials (PANCREOX, CONKO-003, and NAPOLI-1) are too disparate to allow any integrated analysis or direct comparison, and suggests that the results of these trials need individual consideration in the respective clinical situation (10).

Given these difficulties in interpreting the available and partly conflicting evidence, it is not surprising that a wide array of second-line chemotherapy regimens are offered in the real world setting (11). This is not uncontroversial, given the divergent results from phase III trials, and the additional uncertainty on tolerability and outcome when unselected patients are treated outside the frame of a controlled study. In addition, the benefits and drawbacks of second line chemotherapy in patients who have received non-gemcitabine based first line regimes remain largely unproven.

To evaluate the efficacy and safety on second-line chemotherapy in a real world context of patients with APC, a population based retrospective multicenter cohort study was therefore designed. Overall survival, time to treatment failure, hematological toxicity, and unplanned hospitalizations were assessed in a cohort of *all* eligible patients treated at any oncology department in the South East region of Sweden during 2011–2018. In addition, the prognostic impact of clinico-pathological baseline parameters were evaluated, as well as potential outcome differences depending on the type of chemotherapy regimen administered. As the intention was to describe the true real world situation, rather than the efficacy of a certain regimen or sequence of therapy, no selection or exclusion based on the type of first- or second line therapy was undertaken.

## Materials and Methods

### Study Population

A retrospective observational multicenter cohort study in the South East Health Care region of Sweden, covering a population of 1.1 million citizens, was designed. The Swedish health care system is publicly funded, and all cancer treatments in the South East Health Care region are provided at the Departments of Oncology in Jönköping, Kalmar and Linköping.

The digital health care system Cambio Cosmic (version 2.5.16.9531, WEADD TM Sync, EVRY Healthcare Systems AB) was used to identify eligible patients. Inclusion criteria were: Male and female, at least 18 years age, diagnosed pancreatic adenocarcinoma (ICD-10 C25.0–C25.9), registration at any of the three departments of oncology between 1st January 2011 to 31st of December 2017, and the initiation of second-line of palliative chemotherapy before 30th April 2018. Exclusion criteria were histopathology other than adenocarcinoma or secondary metastatic tumor of non-pancreatic origin.

### Ethics

The ethical review board at Linköping University approved the study (Dnr 2018/139-31). A study-specific consent was considered unnecessary given the retrospective design of the trial, and the presumed nearly 100% mortality in this type of cohort.

### Data Extraction

Data were extracted from the medical records and included baseline patient and tumor characteristics, treatment types and duration, follow up of response, hematological toxicity, events of unplanned hospitalization, and baseline biochemistry including plasma albumin and serum CA-19-9. Toxicity was graded in accordance to Common Terminology Criteria for Adverse Events (CTCAE) version 5.0 (12). Two investigators (EG and NB) performed all data collection in tight collaboration to ensure equal interpretation of the medical records. Any disputes were resolved in the team (EG, NB, NE), and followed by a consensus decision. Patients were followed until 30th April 2019 or death, whatever came first.

### Statistics

To compile data, Excel (version 1811) was used. Analyses were performed using SPSS Statistics v25 (IBM, Corp. Armonk NY). The primary outcome was overall survival. Secondary endpoints were time to treatment failure, unplanned hospitalization, and myelosuppression during second-line treatment.

OS was defined as the time interval from the first dose of second-line palliative chemotherapy to death or last follow-up for patients still alive (OS2). Time to treatment failure was defined as the time interval from the first dose of first-line palliative chemotherapy to the last dose of first-line palliative chemotherapy (TTF1) or from the first dose of second-line chemotherapy to the last dose of second-line chemotherapy (TTF2). Median OS2 (mOS2) and TTF2 curves were plotted using Kaplan-Meier survival analysis (13) and compared using the log rank test (14). For OS2 and TTF2, the Cox proportional hazards model (15) was used to estimate hazard ratios (HRs) with 95% confidence intervals (CIs).

Variables entered in the Kaplan-Meier survival analyses and Cox regression analyses were dichotomized as follows: Gender (female vs. male), age (≤ median vs. >median), tumor spread (locally advanced vs. metastasized), ECOG performance status (ECOG PS ≤ 1 vs. >1), serum CA-19-9 (≤ median vs. >median), plasma albumin (<35 vs. ≥35) (reference 35–45 g/L), first-line regimen (monotherapy vs. combination therapy and gemcitabine vs. Gem/NabP), second-line regimen (monotherapy vs. combination therapy and fluoropyrimidine/oxaliplatin doublet vs. fluoropyrimidine single drug regimen), and TTF1 in months (≤ median vs. > median). A *p* < 0.05 (two-sided) was considered statistically significant.

## Results

### Study Population and Treatment Data

**Figure 1:**
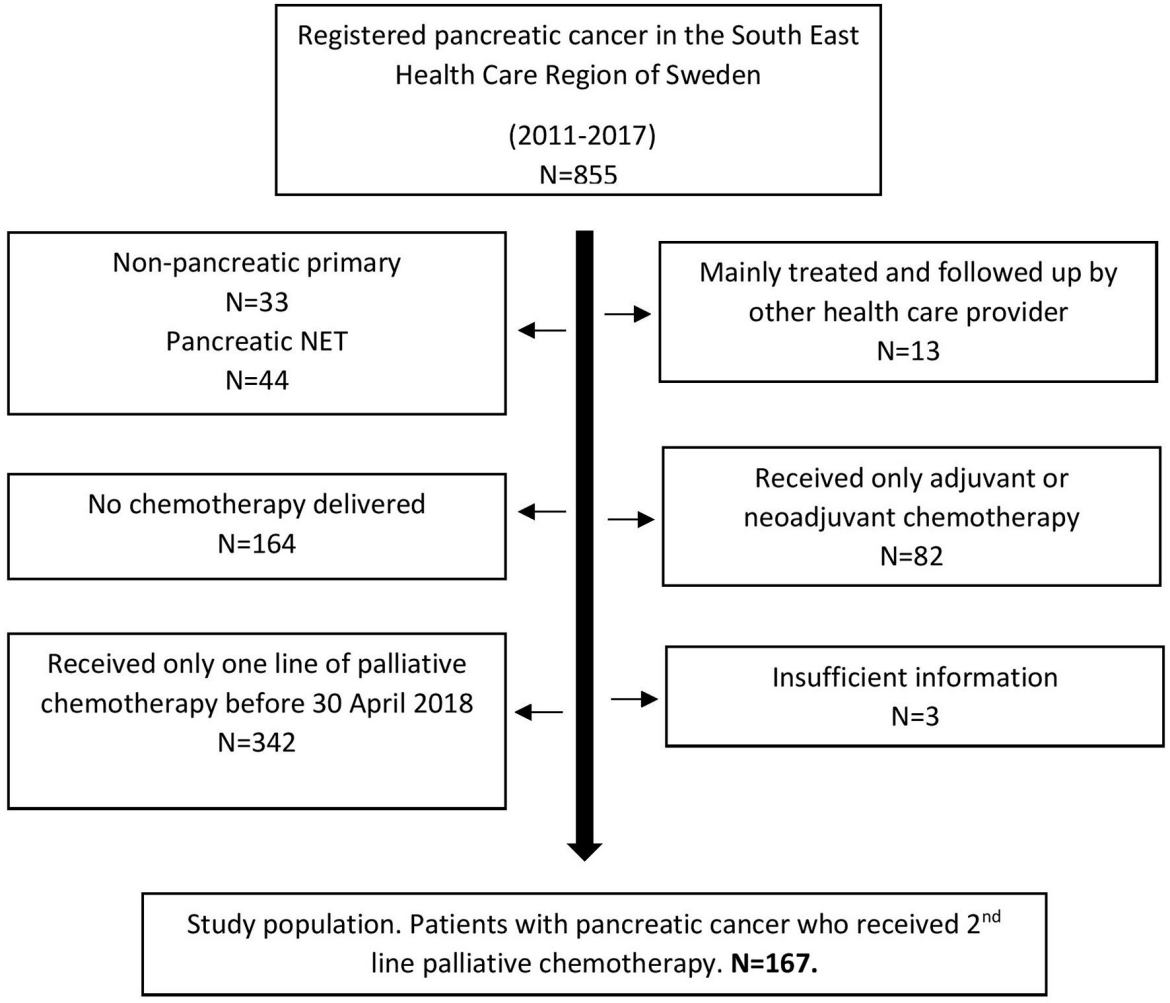
Flow chart study population. NET, Neuroendocrine tumor.

In total, 855 patients with pancreatic adenocarcinoma diagnosed during 2011–2017 were identified (Figure 1). Five hundred and nine patients received first-line palliative chemotherapy, and of these 167 patients (33%) received at least one dose of second-line chemotherapy which means they fulfilled the eligibility criteria and formed the final study population.

Patient and treatment characteristics are presented in Table 1, Supplementary Table 1. The most common first line regimen was gemcitabine monotherapy (*n* = 70, 41.9%) followed by Gem/NabP combination chemotherapy (*n* = 46, 27.5%), gemcitabine/capecitabine (*n* = 14, 8.4%), and FOLFIRINOX (*n* = 14, 8.4%). Regarding second line treatment, 99 (59%) of the patients received a combination of drugs whereas 68 (41%) received a single drug regimen. The two most frequent types of second-line regimens were 5-flourouracil/folinic acid/oxaliplatin (5-FU bolus and infusion based regimens combined) and capecitabine monotherapy, which were delivered to 48 (29%) and 37 (22%) of patients, respectively. When oral and intravenous fluoropyrimidine based regimens (i.e., 5-FU and capecitabine) were grouped, the most common combination was fluoropyrimidine/oxaliplatin which was administered to 66 (40%) patients, whereas the most common single drug regimen was fluoropyrimidine monotherapy (*n* = 51, 31%). Other less common regimens administered are shown in Table 1. Median follow-up time from start of second-line treatment was 5.2 months (interquartile range 2.9–10.4) for the total study population. At last date of follow-up, 163 patients (98%) were dead (Supplementary Table 1).

**Table 1:** Patient and treatment characteristics.

	**Study population**
	***N* = 167 (100%)**
**Gender**	
Female	77 (46.1)
Male	90 (53.9)
**Age (years)**^**a**^	
Median (interquartile range)	67.1 (62.7–72.7)
**Regimen 1st line palliative chemotherapy**	
Gemcitabine	70 (41.9)
Gem/NabP	46 (27.5)
Gem/Cap	14 (8.4)
FOLFIRINOX	14 (8.4)
5-FU/FA/Ox	9 (5.4)
Capecitabine	8 (4.8)
Cap/Ox	3 (1.8)
Others^b^	3 (1.8)
Time to treatment failure under 1st line	*166*
treatment (TTF1) (months) *no*	
Median (interquartile range)	3.65 (2.3–7.2)
ECOG PS^a^, *no*	*122*
0	25 (20.5)
1	70 (57.4)
2	26 (21.3)
3	1 (0.8)
**Extent of tumor spread**^**a**^	
Locally advanced	22 (13.2)
Metastatic disease	145 (86.8)
CA 19-9 (kU/L)^a^, *no*	*103*
Median (interquartile range)	1,550 (200–18,082)
Albumin (g/L)^a^, *no*	*141*
Median (interquartile range)	35.0 (32.5–37.0)
**2nd line palliative chemotherapy**	
Monotherapy	68 (40.7)
Combination therapy	99 (59.3)
**Regimen 2nd line chemotherapy**	
5-FU/FA/Ox	48 (28.7)
Capecitabine	37 (22.2)
Cap/Ox	18 (10.8)
Gemcitabine	16 (9.6)
5-FU/FA	14 (8.4)
5-FU/FA/Iri	11 (6.6)
Gem/NabP	10 (6.0)
Gem/Cap	5 (3.0)
FOLFIRINOX	4 (2.4)
Others^c^	4 (2.4)

a at start of 2nd line chemotherapy.

b 5-FU/FA, Gemcitabine/Oxaliplatin, Gemcitabine/AMG479.

c Gemcitabine/Oxaliplatin, Erlotinib, Dabrafenib/Trametinib.

*Gem/NabP, gemcitabine plus nab-paclitaxel; Gem/Cap, gemcitabine plus capecitabine; FOLFIRINOX, 5-flourouracil, folinic acid, irinotecan, and oxaliplatin; 5-FU/FA/Ox, 5-flourouracil, folinic acid, and oxaliplatin; Cap/Ox, capecitabine plus oxaliplatin; ECOG PS, Eastern Cooperative Oncology Group performance status; 5-FU/FA, 5-flourouracil plus folinic acid; 5-FU/FA/Iri, 5-flourouracil, folinic acid, and irinotecan*.

### Safety and Events of Hospitalization During Second-Line Chemotherapy

During the course of second-line treatment, myelosuppression grade 3–4 was registered in 17 (10%) of the study population. Fifty (30%) of the patients had at least one unplanned hospitalization. Amongst those hospitalized, the median hospital stay was 3 days (interquartile range 1–5 days) (Supplementary Table 2).

### Overall Survival and Time to Treatment Failure

Median OS from date of diagnose was 14.5 months (95% CI 13.0–16.0) and median OS from start of second-line chemotherapy (mOS2) was 5.2 months (95% CI 4.7–5.7) (Table 2). One hundred three patients (63%) discontinued second-line treatment at or before the first planned follow up appointment (Supplementary Table 3). Median TTF2 was 1.9 months (95% CI 1.5–2.2) (Table 3). There were several reasons for early termination of second-line treatment; tumor progression (*n* = 94, 57%), impaired PS (*n* = 59, 36%), toxicity (*n* = 47, 29%), or death (*n* = 7, 4%) (Supplementary Table 3).

**Table 2:** Uni- and multivariable analysis of prognostic factors for OS2.

	***N* (*N* of events)**	**mOS2 (95% CI)**	**HR univariable analysis**	**HR multivariable analysis**
			**(95% CI), *p***	**(95% CI), *p***
Total cohort	167 (163)	5.2 (4.7–5.7)		
**Gender**				
Female	77 (75)	5.2 (4.5–5.9)		
Male	90 (88)	5.1 (4.2–6.0)	1.24 (0.90–1.70) *p =* 0.190	
**Age**				
0–67 years	82 (79)	5.2 (4.3–6.1)		
>67 years	85 (84)	5.1 (4.5–5.7)	1.16 (0.85–1.58) *p =* 0.362	
**Tumor spread**				
Locally advanced	22 (22)	9.4 (5.2–13.7)		
Metastatic	145 (141)	4.9 (4.3–5.6)	1.42 (0.90–2.23) *p =* 0.129	
**ECOG PS**				
0–1	95 (91)	5.8 (4.2–7.3)		
>1	27 (27)	4.2 (3.2–5.1)	1.94 (1.24–3.04) ***p=*** **0.003**	1.55 (0.79–3.02) *p =* 0.202
**CA 19–9 (kU/L)**				
0–1,550	52 (51)	8.5 (5.7–11.3)		
>1,550	51 (50)	4.7 (3.3–6.2)	2.39 (1.55–3.68) ***p<*** **0.001**	2.03 (1.19–3.46**)** ***p=*** **0.009**
**Albumin (g/L)**				
<35	64 (64)	4.0 (3.4–4.7)		
35–47	77 (74)	7.5 (5.1–9.9)	0.39 (0.27–0.57) ***p<*** **0.001**	0.21 (0.11–0.40) ***p<*** **0.001**
**Regimen 1st line**				
Monotherapy	79 (78)	7.0 (5.3–8.6)		
Combination therapy	88 (85)	8.7 (7.0–10.4)	0.81 (0.59–1.10) *p =* 0.172	
**Regimen 1st line**				
Gemcitabine	70 (69)	6.2 (4.8–7.7)		
Gem/NabP	46 (44)	9.2 (6.3–12.1)	0.70 (0.48–1.03) *p =* 0.067	
**Regimen 2nd line**				
Monotherapy	68 (66)	4.8 (3.7–5.9)		
Combination therapy	99 (97)	5.4 (4.5–6.3)	0.79 (0.57–1.08) *p =* 0.135	
**Regimen 2nd line**				
Cap or 5-FU/FA	51 (49)	4.1 (2.9–5.4)		
Cap/Ox or 5-FU/FA/Ox	66 (65)	5.3 (4.7–5.9)	0.88 (0.73–1.07) *p =* 0.189	
**TTF1 (months)**				
0–3.65	83 (81)	4.7 (3.5–5.9)		
>3.65	83 (81)	5.4 (4.8–5.9)	0.84 (0.61–1.14) *p =* 0.253	

*mOS2, Median overall survival in months from start of second line treatment; HR, Hazard ratio; CI, Confidence interval; ECOG PS, Eastern Cooperative Oncology Group performance status; Gem/NabP, gemcitabine plus nab-paclitaxel; Cap, capecitabine; 5-FU/FA, 5-flourouracil plus folinic acid; Cap/Ox, capecitabine plus oxaliplatin; 5-FU/FA/Ox, 5-flourouracil, folinic acid, and oxaliplatin; TTF1, Time to treatment failure under first line treatment. All p values < 0.05 are typed in bold*.

**Table 3:** Uni- and multivariable analysis of prognostic factors for TTF2.

	***N* (*N* of events)**	**mTTF2 (95% CI)**	**HR univariable analysis**	**HR multivariable analysis**
			**(95% CI)**	**(95% CI)**
Total cohort	167 (165)	1.9 (1.5–2.2)		
**Gender**				
Female	77 (76)	1.9 (1.3–2.4)		
Male	90 (89)	1.9 (1.5–2.3)	1.24 (0.91–1.70) *p =* 0.166	
**Age**				
0–67 years	82 (80)	1.6 (1.2–2.1)		
>67 years	85 (85)	1.9 (1.4–2.3)	1.17 (0.86–1.60) *p =* 0.323	
**Tumor spread**				
Locally advanced	22 (22)	3.7 (0.6–6.8)		
Metastatic	145 (143)	1.8 (1.6–2.1)	1.52 (0.97–2.39) *p =* 0.065	
**ECOG PS**				
0–1	95 (93)	2.0 (1.5–2.4)		
>1	27 (27)	1.4 (1.3–1.5)	1.72 (1.11–2.65) ***p=*** **0.013**	2.05 (1.06–3.97) ***p=*** **0.032**
**CA19-9 (kU/L)**				
0–1,550	52 (51)	3.3 (1.4–5.2)		
>1,550	51 (51)	1.4 (1.3–1.6)	2.02 (1.33–3.05) ***p=*** **0.001**	2.03 (1.18–3.49) ***p=*** **0.010**
**Albumin (g/L)**				
<35	64 (64)	1.3 (0.8–1.9)		
35–47	77 (75)	2.6 (1.3–3.8)	0.50 (0.36–0.71) ***p<*** **0.001**	0.22 (0.12–0.40) ***p<*** **0.001**
**Regimen 1st line**				
Monotherapy	79 (79)	3.0 (2.2–3.8)		
Combination therapy	88 (86)	4.3 (3.0–5.5)	0.79 (0.58–1.07) *p =* 0.122	
**Regimen 1st line**				
Gemcitabine	70 (70)	2.7 (1.9–3.5)		
Gem/NabP	46 (45)	4.5 (2.5–6.5)	0.73 (0.50–1.07) *p =* 0.103	
**Regimen 2nd line**				
Monotherapy	68 (67)	1.8 (1.4–2.3)		
Combination therapy	99 (98)	1.9 (1.2–2.5)	0.85 (0.62–1.17) *p =* 0.316	
**Regimen 2nd line**				
Cap or 5-FU/FA	51 (50)	1.8 (1.4–2.3)		
Cap/Ox or 5-FU/FA/Ox	66 (66)	1.8 (1.4–2.2)	0.97 (0.81–1.17) *p =* 0.778	
**TTF1 (months)**				
0–3.65	83 (82)	1.7 (1.4–2.0)		
>3.65	83 (82)	2.3 (1.7–2.9)	0.90 (0.66–1.22) *p =* 0.481	

*TTF2, Time to treatment failure in months from start of second line treatment; HR, Hazard ratio; CI, Confidence interval; ECOG PS, Eastern Cooperative Oncology Group performance status; Gem/NabP, gemcitabine plus nab-paclitaxel; Cap, capecitabine; 5-FU/FA, 5-flourouracil plus folinic acid; Cap/Ox, capecitabine plus oxaliplatin; 5-FU/FA/Ox, 5-flourouracil, folinic acid, and oxaliplatin; TTF1, Time to treatment failure under first line treatment. All p values < 0.05 are typed in bold*.

### Uni- and Multivariable Regression Analysis of Prognostic Factors for OS2 and TTF2

**Figure 2:**
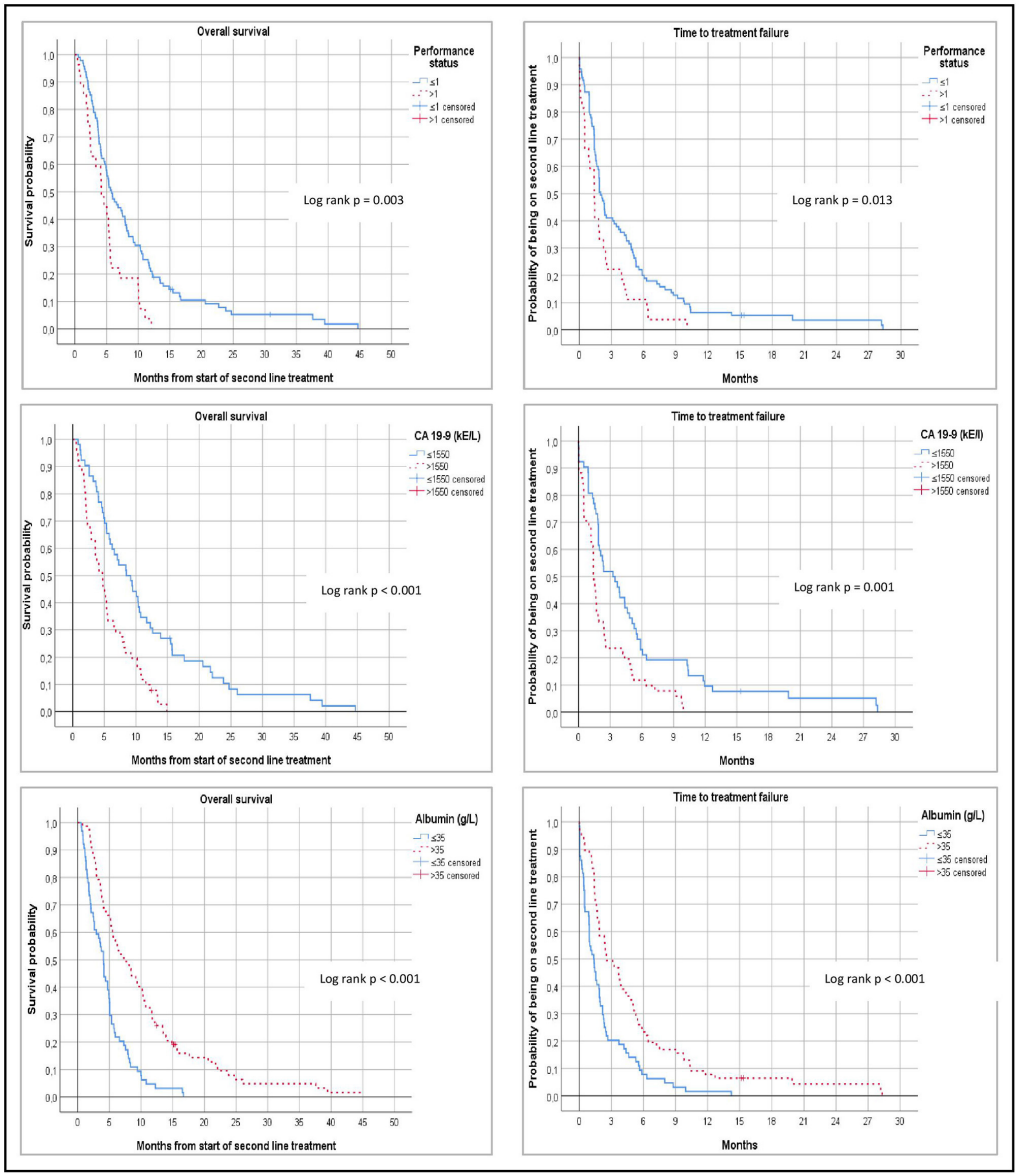
**Left:** Kaplan Meier estimates of overall survival after start of second line chemotherapy to death or last follow-up (OS2). **Right:** Time to treatment failure from start of second-line chemotherapy (TTF2). Log rank test were performed to compare subgroups according to baseline ECOG performance status, levels of serum CA-19-9, and levels of plasma albumin.

Uni- and multivariable analyses of potentially prognostic factors for OS2 and TTF2 are demonstrated in Tables 2, 3, and corresponding Kaplan-Meier graphs are depicted in Figure 2. ECOG PS >1 (HR = 1.94, *p* = 0.003), baseline levels of CA19-9 above median (median = 1,550, HR = 2.39, *p* < 0.001), and baseline plasma albumin equal to or above lower normal limit (lower normal limit = 35 g/L, HR = 0.39, *p* < 0.001) were predictive for OS2. Following multivariable regression analysis, baseline CA-19-9 (HR 2.03, 95% CI 1.19–3.46, *p* = 0.009) and albumin levels (HR 0.21, 95% CI 0.11–0.40, *p* < 0.001) remained independent predictors for OS2, with low CA-19-9 and high albumin representing the most favorable outcome.

In the corresponding analysis of TTF2, ECOG PS >1 (HR = 1.72, *p* = 0.013), and high levels of CA-19-9 (HR = 2.02, *p* = 0.001) were associated with impaired TTF2. On the other hand, normal/high plasma albumin (HR = 0.50, *p* < 0.001) was significantly associated with improved TTF2. These variables remained significantly independent in the multivariable analysis (HR 2.05, 95% CI 1.06–3.97, *p* = 0.032; HR 2.03 95% CI 1.18–3.49, *p* = 0.010; HR 0.22, 95% CI 0.12–0.40, *p* < 0.001).

When comparing TTF2 and OS2 in subgroups of patients receiving single drug vs. multi drug regimen or fluoropyrimidine vs. fluoropyrimidine/oxaliplatin doublet, no significant differences were evident (HR = 0.79, 95% CI 0.57–1.08, *p* = 0.135 and HR = 0.88 95% CI 0.73–1.07, *p* = 0.189, respectively). No significant differences of OS2 and various first line regimens were observed, although there was a trend toward better survival in the first line Gem/NabP vs. the first line gemcitabine monotherapy subgroup (HR = 0.70, 95% CI 0.48–1.03, *p* = 0.067). Neither did metastatic vs. locally advanced tumor, type of treatment in first-line, or duration of first-line chemotherapy emerge as statistically significant prognostic factors (Tables 1, 2).

On CA-19-9 and age, the cutoff levels chosen were equal to the respective median values in the present population. This was considered the most optimal and pragmatic approach, given the lack of established or validated cutoffs in the literature. However, in complementary analyses, alternative and simpler rounded cutoffs of serum CA-19-9 </> 1,500 and age </> 70 were adopted, revealing similar results (HR = 2.19, 95% CI 1.43–3.36, *p* < 0.001 and HR = 1.19, 95% CI 0.86–1.64, *p* = 0.301, respectively).

## Discussion

This population-based study, covering all patients with APC in the South East health care region of Sweden under a period of 7 years, demonstrates poor outcomes in patients receiving second-line palliative chemotherapy. Generally, treatment duration and overall survival in this cohort were restricted to about 2 and 5 months, respectively. Baseline serum CA-19-9, plasma albumin, and performance status were independent prognostic factors and should be further evaluated as tools for risk stratification and treatment guidance.

The proportion of patients receiving second-line chemotherapy in our study was 33% which is consistent with reports from other countries (16). The most common type of regimen was a combination of fluoropyrimidine and oxaliplatin doublet, and the second most common was a fluoropyrimidine single drug regimen. Notably, mOS2 and mTTF2 were similar regardless of regime when comparing the most common type of single drug regimen (i.e., fluoropyrimidine) with the most common multidrug regimen (fluoropyrimidine + oxaliplatin). Similarly, when grouping and comparing all single drug vs. all multidrug regimens, no survival benefit was revealed.

As this was a population-based real world study and not a controlled trial, no selections, or exclusions due to age, comorbidities, performance status, laboratory test results, and/or type of first and second line therapies were undertaken. While a majority received single drug regimens as first line treatment, most commonly gemcitabine monotherapy, only 36% received any of the most potent first line combination regimens (Gem/NabP or FOLFIRINOX). Although this study was not intended to compare various first line regimens, a non-significant trend toward better OS2 in the subgroup of patients who received Gem/NabP rather than gemcitabine monotherapy in the first line situation was observed. Previous studies assessing the real world outcome of first line Gem/NabP, FOLFIRINOX, and gemcitabine monotherapy in APC reported overall survival measures of ~10, 10, and 7 months, respectively (17, 18). While this indicates that the outcomes observed in the current study might have been be slightly improved should Gem/NabP have been more widely used in the first line setting, it remains unclear whether Gem/NabP as second line therapy following failure of e.g., first line FOLFIRINOX would be superior to milder regimens such as gemcitabine monotherapy. Again, no significant differences in survival following second line gemcitabine or Gem/NabP was present in the present cohort, but the limited usage of FOLFIRINOX in the first line situation precludes conclusions of specific treatment sequences such as FOLFIRINOX followed by Gem/NabP.

Obviously, the wide array of first line regimens given makes interpretation of second line data more difficult. Nevertheless, we believe that the results of this study add highly relevant information and complement the results of controlled trials, since they reflect the true real world situation and not only highly selected patients who were eligible for certain types of regimens in the first and second line settings.

Given the lack of unequivocal evidence on the optimal treatment strategy and sequence of treatments in APC, it is not surprising that a number of different regimens were administered in the second-line situation. This makes direct comparisons with other studies difficult since they often focus on only one or two specific second-line treatments. However, two similar retrospective studies on real world unselected patients with pancreatic cancer reported mOS2 of 5.1 months and mOS2 5.3 months following start of second-line therapy (19, 20). These numbers are almost identical to the mOS2 of 5.2 months observed in the present patient cohort. Two other observational studies included patients with all forms of second-line regimens, but first-line treatment was restricted to gemcitabine based regimens only (21, 22). These publications report mOS2 measures ranging from 4.5 to 7.3 months.

In the present cohort, TTF2 (i.e., time to treatment failure from start of second-line therapy) was 1.9 months. TTF2, rather than progression free survival (PFS), was selected as key secondary endpoint since there was no standard schedule for follow up CT scans. Furthermore, many patients (63%) discontinued treatment before or at the first follow up appointment due to clinical progression and/or impaired performance status, which would make PFS data uncertain. While many other studies fail to report TTF2, the TTF2 measures reported in the randomized second-line NAPOLI-1 trial (5) were 1.4, 1.7, and 2.3 months in the respective treatment arms (FF, nal-irinotecan, FF/nal-irinotecan) which are comparable with our data.

Our study confirms that the prognosis for patients with pancreatic cancer treated beyond first-line chemotherapy remains dismal. Due to the high rate of patients discontinuing the treatment following less than a few months of treatment, it is reasonable to argue that a majority of the patients who commence second-line chemotherapy have no or very limited benefit of the treatment. This underlines the need for prognostic and predictive factors in order to offer treatment to those who are likely to benefit from the treatment, while avoiding treatment to those where no benefit is expected.

Regression analyses were performed in order to define potentially prognostic baseline parameters. In line with previous reports (19, 20, 22–24), no prognostic impact of age or gender was evident in terms of mOS2 and TTF2. Neither was tumor burden (locally advanced vs. metastatic disease) a statistically significant prognostic parameter. However, ECOG performance status (≤ 1 vs. >1) had a significant impact on the risk for death and treatment failure in univariable regression analyses. Following multivariable regression analysis, the statistical significance remained for TTF2 but not for mOS2. Estimated mTTF2 was 2.0 and 1.4 months in patients with ECOG performance status of ≤ 1 and >1, respectively. These results, together with other publications (16, 21–23), imply that ECOG performance status >1 predicts a poor outcome with short time to treatment failure and progression, and raise uncertainty whether second-line chemotherapy has any role in this type of patients.

Beside ECOG performance status, plasma albumin, and serum CA-19-9 at start of second-line treatment were found to independently predict both OS2 and TTF2 in multivariable regression analyses. In general, mOS2 and mTTF2 were doubled in patients with favorable baseline albumin and CA-19-9 levels, respectively. Since there is no generally established high/low cut-off for serum CA-19-9, we considered that the median value was the most reasonable cut off limit. While there is at least one previous publication indicating a prognostic value of CA-19-9 in second-line setting (19), to our knowledge baseline albumin has previously not been assessed as a prognostic marker in the second-line situation. Our findings imply that serum baseline CA-19-9 and albumin should be routinely tested in clinical trials on APC second-line therapy, and should be further evaluated as prognostic tools.

The incidence of grade 3–4 hematological toxicity and unplanned hospitalizations was low, and the most common reason for terminating second-line therapy was not treatment related complications but disease progression and/or impaired performance status. Given the retrospective nature of the study, it was not possible to collect data on “subjective” adverse events such as fatigue, gastrointestinal symptoms, and neurotoxicity. Unplanned hospitalizations were therefore selected as a proxy parameter for severe adverse events. Naturally, the short treatment durations meant that the exposure to the drugs was limited, but nevertheless, these results indicate that APC second line therapy is usually well-tolerated in the real world context.

The main strength of the present study is the real world approach meaning that *all* patients with APC treated at any of the hospitals in the region under a period of 7 years and given at least one dose of second-line palliative chemotherapy were included. A significant effort was undertaken to register all relevant information on outcome, hematological toxicity, and unplanned hospitalizations, both at baseline and during the treatment course. This means that the present study is highly relevant for defining the expected outcome of second-line therapy, and for exploring potentially prognostic biomarkers in real world unselected patients, which may or may not differ from what is evident in patients included in controlled prospective trials.

The main weaknesses naturally mirror the main strength; the retrospective unselected inclusion and the wide array of treatment regimens given means that the study design is not optimal to define the efficacy of a certain kind of regimen. A majority of patients were treated before combination regimens such as FOLFIRINOX and Gem/NabP were generally established, and it is possible that today's wider usage of combination regimens in the first line setting may improve the prospects for second line therapies in future. In addition, many European countries including Sweden do not offer nal-irinothecan plus fluorouracil combination chemotherapy within the public health care. This means that the present results should be cautiously interpreted, and not necessarily reflect the outcome in a Northern American real world context (where nal-irinothecan is more commonly provided).

## Conclusion

The present study provides real world evidence on second line chemotherapy in APC. The expected survival is short regardless the type of chemotherapy regimen given, with median time to treatment failure and overall survival limited to about 2 and 5 months, respectively. High baseline levels of CA-19-9, low levels of plasma albumin, and ECOG performance status >1 are negative prognostic factors, and it is questionable what role (if any) current second-line chemotherapy has in these groups of patients. Further trials are needed to define tools for risk stratification and therapeutic guidance.

## Data Availability Statement

The datasets generated for this study are available on reasonable request to the corresponding author.

## Ethics Statement

The studies involving human participants were reviewed and approved by Ethics Committee Linköping University. Written informed consent for participation was not required for this study in accordance with the national legislation and the institutional requirements.

## Author Contributions

EG, NB, EÅ-L, HB, and NE designed the study. EG and NB collected and organized the data. EG, NB, HB, MF, and NE analyzed the data. All authors interpreted and discussed the data, were major contributors to the manuscript, and read and approved the final manuscript.

## Conflict of Interest

The authors declare that the research was conducted in the absence of any commercial or financial relationships that could be construed as a potential conflict of interest.
