# Supplementary material for: Real World Evidence on Second-Line Palliative Chemotherapy in Advanced Pancreatic Cancer
Source: Front Oncol. 2020 Jul 27;10:1176. doi: 10.3389/fonc.2020.01176 (PMC7397908; doi:10.3389/fonc.2020.01176)
Supplement: Supplementary file 1 [file Data_Sheet_1.docx]

**Gränsmark, Supplementary material**

**Supplementary table 1**. Complementary patient and treatment characteristics

|  | **Study population**  **N=167 (%)** |
| --- | --- |
| BMI (kg/m^2^)^1^ *no*  Median (interquartile range) | *120*  23.1(20.7-25.7) |
| Smoking^1^, *no*  Never smoked  Former smoker  Current smoker | *139*  63 (45.3)  47 (33.8)  29 (20.9) |
| Previous treatment  Radiotherapy  Yes  Target primary tumour  Target metastasis  No  Pancreatic resection  Yes  No  Neoadjuvant chemotherapy  Yes  No  Adjuvant chemotherapy  Yes  No | 4 (2.4)  6 (3.6)  157 (94.0)  46 (27.5)  121 (72.5)  10 (6.1)  154 (93.9)  37 (22.2)  130 (77.8) |
| Site of metastasis^1, 2^  Liver  Lung  Peritoneal cavity  Lymph nodes  Bone  Brain  Other | 94 (64.8)  55 (37.9)  35 (24.1)  27 (18.6)  10 (6.9)  0  3 (2.1) |
| Follow-up (months) ^3^, *no*  Median (interquartile range) | *dead 163, alive 4*  5.2 (2.9-10.4) |

^1^ at start of 2nd line chemotherapy
^2^ Non-exclusive i.e. cases may have multiple metastatic sites

^3^ from start of 2nd line treatment
Abbreviations: BMI = Body mass index

| **Supplementary table 2.** Toxicity during ongoing second line chemotherapy | | | |
| --- | --- | --- | --- |
|  | |  | N (valid percent) |
| Myelosupression grade 3-4^1^ | | Yes | 17 (10.2%) |
|  | | No | 150 (89.8%) |
| Hospitalised during 2nd line treatment | | Yes | 50 (30.3%) |
|  | | No | 115 (69.7%) |
|  | | MD | 2 |
| Number of nights hospitalised^2^, median (quartiles) | | 3.0 (1.0-5.0) |  |
| Proportion of time during treatment spent hospitalised^2^, median (quartiles) | | 3.5% (1.2 -8.7%) |  |
| ^1^ according to Common Terminology Criteria for Adverse Events  ^2^ among the patients who were hospitalised during 2nd line treatment Abbreviations: MD = missing data | | |  |
|  |  | |  |
|  |  | |  |

| **Supplementary Table 3.** Treatment discontinuation and OS from primary diagnose | | |
| --- | --- | --- |
|  |  | N (valid percent) |
| Median OS from primary diagnose | 14.5 months | N=163 |
|  | (CI 95% 13.0-16.0) | N of events=159 |
| 2nd line continued following first follow up | Yes | 62 (37.6%) |
|  | No, discontinuation before follow up | 61 (37.0%) |
|  | No, discontinuation at first follow up | 42 (25.5%) |
|  | MD | 2 |
| Reasons for ending 2nd line treatment^1^ | Progression | 94 (57%) |
|  | Impaired general appearance | 59 (36%) |
|  | Toxicity | 47 (29%) |
|  | Death | 7 (4%) |

^1^ more than one reason allowed
Abbreviations: CI = confidence interval; OS = overall survival; MD = missing data

| **Supplementary Table 4.** Myelosuppression during ongoing 2nd line therapy | | | | |  |
| --- | --- | --- | --- | --- | --- |
| **Grade**^1^ | **None** | **1** | **2** | **3** | **4** |
| Anemia | 46 (27.5%) | 84 (50.5%) | 34 (20.4%) | 2 (1.2%) | 1 (0.6%) |
| Leukopenia | 134 (80.2%) | 10 (6.0%) | 20 (12.0%) | 2 (1.2%) | 1 (0.6%) |
| Neutropenia | 134 (80.2%) | 4 (2.4%) | 16 (9.6%) | 8 (4.8%) | 5 (3.0%) |
| Thrombocytopenia | 110 (65.9%) | 49 (29.3%) | 4 (2.4%) | 4 (2.4%) | 0 |

^1^ according to Common Terminology Criteria for Adverse Events
